# Gender Disparities in Shoulder Pain and Shoulder Surgery: A Current Concepts Review

**DOI:** 10.3390/jcm15051886

**Published:** 2026-03-01

**Authors:** Mohammad Daher, Tarishi Parmar, Peter Boufadel, Ziad Zalaquett, Mohamad Y. Fares, Joseph A. Abboud

**Affiliations:** 1 Division of Shoulder and Elbow Surgery, Rothman Orthopaedic Institute, Philadelphia, PA 19107, USAabboudj@gmail.com (J.A.A.); 2 Department of Orthopaedics, University of Pittsburgh Medical Center, Pittsburgh, PA 15213, USA

**Keywords:** gender, disparity, shoulder arthroplasty, rotator cuff arthropathy, rotator cuff repair, instability

## Abstract

Shoulder pain and shoulder surgery are increasingly prevalent and encompass a broad spectrum of pathologies, including rotator cuff disease, glenohumeral osteoarthritis, and shoulder instability. Growing evidence suggests that gender-related factors influence disease presentation, patient-reported outcomes, and postoperative recovery; however, these effects remain inconsistently reported across the literature. This current concepts review synthesizes available evidence on the influence of gender on pre-operative characteristics, non-operative management, and postoperative outcomes following common shoulder procedures, including rotator cuff repair, anatomic and reverse shoulder arthroplasty, and surgical stabilization for instability. A comprehensive literature search of PubMed, the Cochrane Library, and Google Scholar was performed for studies published through October 2025, with outcomes assessed using validated instruments such as the Western Ontario Rotator Cuff Index, American Shoulder and Elbow Surgeons score, Constant–Murley score, Simple Shoulder Test, Visual Analog Scale, and Shoulder Pain and Disability Index. Across shoulder pathologies, female patients consistently demonstrated worse pre-operative functional scores, higher pain levels, and greater perceived disability despite similar structural disease severity. Postoperatively, both genders experienced meaningful clinical improvement; however, females often reported higher early postoperative pain and lower absolute functional outcomes, particularly following shoulder arthroplasty for glenohumeral osteoarthritis and surgical treatment of multidirectional instability. In contrast, outcomes following rotator cuff repair and anterior instability stabilization were largely comparable between genders. Recognition of these gender-related differences is essential for individualized patient counseling, expectation setting, and optimization of management strategies, and highlights the need for future studies with robust gender-disaggregated analyses.

## 1. Introduction

The impact of shoulder pain is powerful as it affects all aspects of the patient’s quality of life [1,2]. In fact, this problem has a variety of detrimental effects on daily life with some people becoming unable to work, others unable to provide care, and many people lose out on leisure activities that are crucial to their physical and mental well-being [1,2]. Traditional symptoms include pain and weakness when doing activities and fragmented sleep due to nocturnal pain as well as instability [2,3].

The patient population suffering from various shoulder pathologies is diverse, including males and females between the ages of 15 and 80, office workers and laborers, sedentary people, and physically active people with a wide range of occupations [4]. Therefore, to increase the percentage of “successful” management, a lot of attention has been paid to the biological and technical aspects of this problem. However, treating structural issues on its own does not necessarily result in the desired therapeutic outcomes from the patient’s perspective which is why factors that are specific to the patient such as gender have a role in affecting patient-reported outcomes (PROMs) post-operatively [1,2]. In fact, it is crucial to look into how gender may affect outcomes of shoulder surgery since upper-limb function affects daily activities differently in males and females.

Although the influence of gender on the management of shoulder pathologies is receiving greater attention, the focus is still lacking. Therefore, the purpose of this review is to study the effect of gender on the various shoulder-related issues and their management.

## 2. Data Collection

A comprehensive literature search was conducted through PubMed, Cochrane Library, and Google Scholar (pages 1–20) with searches updated through October 2025. Studies evaluating sex- and gender-based differences in shoulder pathology and surgical outcomes, including rotator cuff pathology, glenohumeral osteoarthritis (GHOA), and shoulder instability were identified. Boolean operators were applied to combine the following keywords: “gender,” “sex,” “shoulder,” “arthroplasty,” “replacement,” “rotator cuff,” “instability,” and “glenohumeral osteoarthritis.” Additional studies were identified by reviewing reference lists from relevant articles and performing supplementary online searches. The review focused on studies evaluating gender-based differences in patients undergoing conservative management for the listed diagnoses or operative management including rotator cuff repair (RCR), anatomic and reverse shoulder arthroplasty (aTSA, RSA), and surgical stabilization procedures for shoulder instability. The data was extracted by one researcher, and the article selection was verified by a different researcher.

## 3. Rotator Cuff Pathology (Figure 1)

### 3.1. Effect of Gender on Pre-Operative Characteristics (Table 1)

The severity and functional status of patients with rotator cuff pathologies vary between males and females. A cross-sectional study by Razmjou et al. investigated the differences in pre-operative characteristics in 85 males and 85 females who were candidates for rotator cuff surgery using the Western Ontario Rotator Cuff (WORC) index [5]. While there were no significant differences in both radiological and intra-operative assessments of the level of rotator cuff pathology, females were reported to have higher levels of disability with poorer shoulder function and quality of life [5]. The authors reported females to have lower levels of strength with decreased active flexion and abduction as well as decreased combined pain-free range of motion compared to males [5]. This coincides with another study by Bassey et al. that reported lower level of abduction as well as increased hesitancy in shoulder movement beyond painful range in females compared to males [6].

**Figure 1:**
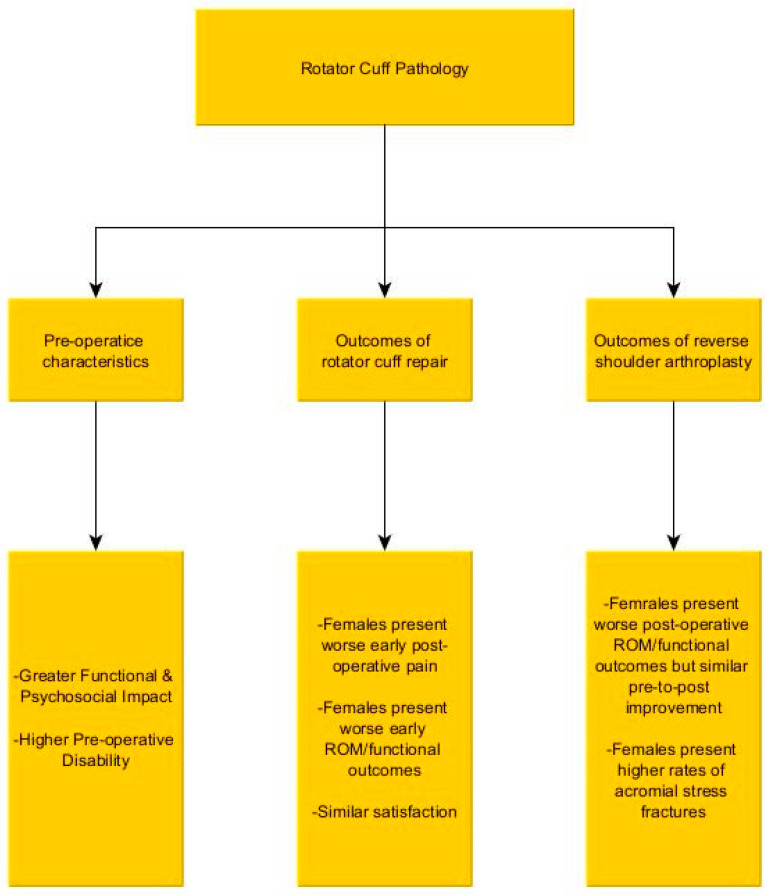
Summary of the literature regarding the impact of gender on rotator cuff pathology, and its management.

**Table 1:** Gender differences in rotator cuff pathology and surgery.

Effect of Gender on Rotator Cuff Pre-operatively	Bassey et al. 1989 [6]	-Lower level of abduction and increased hesitancy in shoulder movement in females.
	Romeo et al. 1999 [7]	-Older age in females was associated with a higher Constant–Murley score, Simple Shoulder Test score, and lower level of reported disability when compared to males
	Razmjou et al. 2009 [5]	-No radiologic or intra-operative difference in the level of rotator cuff pathology. -Poorer shoulder function and quality of life in females. -Lower level of strength and decreased active flexion, abduction, and pain free range of motion in females. -Increased interference and limitations of participation in social activity in females. -Modified post-operative expectations. -Increased emotional disturbance in females
	Maher et al. 2017 [8]	-Lower pre-operative Flex-SF score and higher mean pain score in females.
	Gibson et al. 2019 [9]	-Females have a lower Western Ontario Rotator Cuff-lifestyle subscale score.
	Gruber et al. 2023 [10]	-Females have significantly more fatty degeneration of the rotator cuff muscles -Both showed the same progression of cuff tear arthropathy
Effect of Gender on Rotator Cuff Repair Post-operatively	Watson et al. 2002 [11]	-A higher proportion of women reported improvements in activities of daily living post-operatively.
	Feng et al. 2003 [12]	-No effect of gender on post-operative outcomes.
	O’Holleran et al. 2005 [13]	-No effect of gender on patient satisfaction following surgery.
	Charousset et al. 2008 [14]	-Female gender was shown to be a negative predictive factor for the improvement of the Constant–Murley score post-operatively.
	Oh et al. 2009 [15]	-Lower Simple Shoulder Test in Females.
	Razmjou et al. 2011 [16]	-Higher disability in females post-operatively -Females with lower strength pre-operatively had lower satisfaction following surgery. -Females involved in work-related injuries and receiving benefits from the compensation board had lower post-operative satisfaction compared to women who were not involved in such injuries.
	Cho et al. 2015 [17]	-Higher mean visual analog scale scores in females 2 weeks post-operatively, but no difference at 6 weeks, 3, 6, and 12 months post-operatively. -Lower mean forward flexion at 6 weeks post-operatively in females. -Lower mean external rotation at 6 weeks and 3 months post-operatively in females.
	Daniels et al. 2019 [18]	-Higher mean visual analog scale scores in females 2, 6 weeks, and 3 months post-operatively. -Higher consumption of pain medication in females 2 weeks post-operatively. -Greater overall change in mean visual analog scale scores from pre-operatively to 1 year post-operatively in females. -Lower American shoulder and elbow surgeons score in females 3 months post-operatively.
	Sabo et al. 2021 [1]	-No significant difference in mean post-operative visual analog scale scores. -No significant difference in post-operative satisfaction.
	Zeng et al. 2024 [19]	Women had statistically significantly poorer functional outcome and pain scores at 1 and 2 years after rotator cuff repair. They also experienced less improvement in outcome scores throughout the postoperative period. Women had statistically significantly lower rates of PASS attainment at 2 years after rotator cuff repair.
	Harley et al. 2025 (Systematic review) [20]	Female and male patients showed no significant difference in retear rate following rotator cuff repair.
Effect of Gender on Rotator Cuff Repair Post-operatively	Watson et al. 2002 [11]	-A higher proportion of women reported improvements in activities of daily living post-operatively.
	Feng et al. 2003 [12]	-No effect of gender on post-operative outcomes.
	O’Holleran et al. 2005 [13]	-No effect of gender on patient satisfaction following surgery.
	Charousset et al. 2008 [14]	-Female gender was shown to be a negative predictive factor for the improvement of the Constant–Murley score post-operatively.
	Oh et al. 2009 [15]	-Lower Simple Shoulder Test in Females.
	Razmjou et al. 2011 [16]	-Higher disability in females post-operatively -Females with lower strength pre-operatively had lower satisfaction following surgery. -Females involved in work-related injuries and receiving benefits from the compensation board had lower post-operative satisfaction compared to women who were not involved in such injuries.
	Cho et al. 2015 [17]	-Higher mean visual analog scale scores in females 2 weeks post-operatively, but no difference at 6 weeks, 3, 6, and 12 months post-operatively. -Lower mean forward flexion at 6 weeks post-operatively in females. -Lower mean external rotation at 6 weeks and 3 months post-operatively in females.
	Daniels et al. 2019 [18]	-Higher mean visual analog scale scores in females 2, 6 weeks, and 3 months post-operatively. -Higher consumption of pain medication in females 2 weeks post-operatively. -Greater overall change in mean visual analog scale scores from pre-operatively to 1 year post-operatively in females. -Lower American shoulder and elbow surgeons score in females 3 months post-operatively.
	Sabo et al. 2021 [1]	-No significant difference in mean post-operative visual analog scale scores. -No significant difference in post-operative satisfaction.
	Zeng et al. 2024 [19]	Women had statistically significantly poorer functional outcome and pain scores at 1 and 2 years after rotator cuff repair. They also experienced less improvement in outcome scores throughout the postoperative period. Women had statistically significantly lower rates of PASS attainment at 2 years after rotator cuff repair.
	Harley et al. 2025 (Systematic review) [20]	Female and male patients showed no significant difference in retear rate following rotator cuff repair.
Effect of Gender on Reverse Shoulder Arthroplasty Post-operatively	Wong et al. 2017 [21]	-Females had worse SF-12 physical health composite score and American shoulder and elbow surgeons score function subscale. -No difference between genders in SF-12 mental health composite score, American shoulder and elbow surgeons score pain subscale, visual analog scale score for pain, and post-operative opiate usage. -No difference in post-operative range of motion between males and females.
	Friedman et al. 2018 [22]	-Females had worse simple shoulder test score, constant score, University of California Los Angeles shoulder score, American shoulder and elbow surgeons score, and shoulder pain and disability index. -No difference in pre-operative to post-operative improvement of these scores between males and females. -Females had worse abduction, passive external rotation, and forward flexion. -No difference in pre-operative to post-operative range of motion improvement between males and females.
	Chelli et al. 2022 [23]	-No difference in post-operative complications and implant survivorship between males and females.
	Nielsen et al. 2022 [24]	-No difference in post-operative Western Ontario osteoarthritis of the shoulder score between males and females
	Mahendraraj et al. 2023 [25]	-Female gender was a predictive factor for post-operative acromion and scapular spine stress fracture.

Moreover, females reported increased interference and limitations in social activity participation in comparison to males [5]. Females were also dissimilar to males in their expectations for improved ability of carrying out the normal activities of daily living, which included sleeping and performing routine tasks, such as dressing, styling hair, and performing overhead movements [5]. Similarly, Gibson et al. found females to have poorer WORC-lifestyle subscale scores, suggesting females may experience greater functional impacts to specific lifestyle elements than males [9]. These limitations may relate to the aforementioned inferior pain-free range of motion. Razmjou et al. also demonstrated in another cross-sectional study that females with rotator cuff pathologies had more frustration, depression and worry attributed to their shoulder pathology, suggesting increased emotional disturbance in females compared to males [26].

Furthermore, Maher et al. performed a prospective cohort study of 1383 patients undergoing RCR to explore variables affecting shoulder pain and function [8]. The authors found that males had significantly higher preoperative Flex-SF functional scores and significantly lower preoperative mean pain scores compared with females [8]. However, while statistically significant, the difference in Flex-SF scores may not be of clinical significance. The intersection of age and gender in relation to rotator cuff pathology was also explored by Romeo et al. [7]. The authors demonstrated that the effect of aging on disability differs between males and females, with increased aging associated with increased Constant–Murley and Simple Shoulder Test (SST) scores and reduced level of reported disability in females, but not in males [7].

Gruber et al. performed a retrospective cohort analysis of 342 shoulders (257 females and 85 males) that underwent RSA, in aim of assessing morphological irregularities and the natural progression of GHOA in males and females using preoperative X-rays, computed tomography and magnetic resonance imaging [10]. The authors found that females showed significantly more fatty degeneration of the rotator cuff muscles in comparison to males [10]. However, this may be attributed to the significantly greater mean age of female patients undergoing RSA compared to males (74.4 years vs. 70.1 years) in this study. Both genders showed the same progression of cuff tear arthropathy as well [10].

Exploring the role of gender in rotator cuff pathologies is important to better be able to understand and address the factors that impact the lives of patients the most and thus improve treatment for both males and females. While the current literature presents females as having higher levels of disability, further research is required to help understand the latter as well as the relationship between gender and preoperative characteristics.

### 3.2. Effect of Gender on Post-Operative Outcomes of RCR (Table 1)

Gender’s impact on clinical and radiological outcomes after RCR has received less attention, yet it holds significant influence. Identifying the differences in outcome between males and females following RCR is of primordial importance to improve models of care and offer recommendations for more effective interventions. Several studies have explored how gender affects clinical and radiological outcomes following RCR.

#### 3.2.1. Effect of Gender on Post-Operative Pain Following RCR

A prospective analysis of 80 patients by Cho et al. showed significantly higher mean visual analogue scale (VAS) scores in females 2 weeks after surgery; however, follow-up at 6 weeks, 3 months, 6 months, and 12 months post-op showed no significant difference in mean VAS scores between males and females (despite females having higher scores) [17]. However, Daniels et al. showed higher VAS scores in females compared to men, with results being statistically significant at 2 weeks, 6 weeks, and 3 months postoperatively [18]. Additionally, females were more likely to use narcotic pain medication at 2 weeks following RCR, and had a greater overall change in the mean VAS score at 1 year postoperatively [18]. Similar results were reported by Zeng et al. in their study [19]. On the other hand, a study involving 148 participants by Sabo et al. failed to demonstrate a significant difference between males and females in postoperative VAS scores at any time point [1]. While there is a tendency for higher pain scores in females, the current research remains ambiguous, and no conclusions can therefore be drawn on this matter.

#### 3.2.2. Effect of Gender on Post-Operative Range of Motion (ROM) and Strength Following RCR

Cho et al. found that females exhibited lower mean forward flexion at the 6-week follow-up, as well as lower mean external rotation at both 6 weeks and 3 months postoperatively [17]. Razmjou et al.’s cohort additionally showed higher disability in females after RCR, with disability being associated with pain-limited ROM, participation limitation, and strength [16]. Females also showed lower shoulder function as evaluated by the American Shoulder and Elbow Surgeons (ASES) score on 3 months follow-up [18]. Oh et al. also showed lower SST scores in females [15]. Moreover, female gender was a negative predictor of improvement of the Constant score, as demonstrated by Charousset et al. [14] Similar results were reported by Zeng et al. in their study [19]. However, Feng et al.’s retrospective analysis of 1120 shoulders showed no significant influence of gender on postoperative outcome [12]. As for the rate of retears after RCR, a recent systematic review by Harley et al. reported no difference between males and females in the rate of retear [20].

#### 3.2.3. Effect of Gender on Post-Operative Satisfaction Following RCR

In contrast to men, females who had lower strength reported lower satisfaction following surgery [16]. Additionally, among females, those involved in work-related injuries and receiving benefits from the compensation board reported lower satisfaction compared to females who were not involved in such injuries. However, no significant impact of work-related injuries on satisfaction was observed in males [16]. Nonetheless, Sabo et al. reported no significant difference in patient satisfaction at 12-month follow-up between males and females, with most patients being satisfied with their RCR surgery [1]. O’Holleran et al. also showed no effect of gender on patient satisfaction following surgery [13]. Furthermore, in an unexpected finding, Watson and Sonnabend’s study revealed that a higher proportion of females reported improvements in activities of daily living after undergoing RCR [11].

In conclusion, additional research is needed to evaluate the variations in outcomes between genders following RCR. This further investigation will contribute to the existing limited knowledge and assist in establishing the expectations of both healthcare providers and patients regarding postoperative recovery patterns.

### 3.3. Effect of Gender on Post-Operative Outcomes of RSA (Table 1)

One of the main indications for RSA is rotator cuff arthropathy [22]. Like in RCR, gender difference can have an impact on the post-operative outcome of RSA. Many studies analyzed this gender effect on post-operative outcomes of RSA but there are few that only focused on patients with rotator cuff tear with or without arthropathy.

#### 3.3.1. Effect of Gender on Post-Operative Complications Following RSA

Contradictory results exist about the effect of gender on post-operative complications. On one hand, Chelli et al. [23] showed that after stratification for the indication of RSA, there was no difference in the post-operative complications and survivorship of the implant between males and females. On the other hand, the female gender was shown to be a predictive factor of scapular spine and acromion stress fractures [25]. The latter can be explained by the high prevalence of osteoporosis, diminished bone quality, patient fragility, vitamin D deficiency, and post-menopausal decrease in bone density [25].

#### 3.3.2. Effect of Gender on Post-Operative Functional Scores Following RSA

It was shown that at the latest post-operative follow-up after RSA for rotator cuff-related indications, females had worse SST score, constant score, University of California Los Angeles (UCLA) shoulder score, ASES score, and shoulder pain and disability index (SPADI) [22]. Moreover, the findings remained the same even after controlling for age. However, the pre-operative to post-operative improvement in each of these scores did not differ between males and females [22]. Worse post-operative functional scores (ASES function subscale, and SF-12 physical health composite score) in females were as well reported by Wong et al. [21] while showing no difference in the ASES pain subscale, SF-12 mental composite score, and visual analog scale score for pain as well as opiate usage. Nevertheless, Nielsen et al. did not report any difference in the Western Ontario osteoarthritis of the shoulder (WOOS) score between gender [24].

#### 3.3.3. Effect of Gender on Post-Operative Range of Motion Following RSA

Friedman et al. [22] reported better post-operative range of passive external rotation and abduction. These findings remained significant after controlling for age. Forward flexion was significantly better in males [22]. However, the improvement in ROM post-operatively did not differ between these two gender [22]. To further support this finding, Wong et al. [21] did not report any difference in the post-operative ROM between males and females.

## 4. Glenohumeral Osteoarthritis (Figure 2)

### 4.1. Effect of Gender on Pre-Operative Characteristics (Table 2)

Pre-operative characteristics of patients with GHOA differ between males and females with respect to age at onset, prevalence, underlying etiology, and baseline disease characteristics. Epidemiologic evidence suggests that gender influences both the timing of disease onset and the pathways leading to degenerative changes of the glenohumeral joint. In a study evaluating the prevalence of GHOA among patients referred for shoulder radiographs, GHOA was commonly identified in both genders, with increasing prevalence observed with advancing age, particularly among female patients [27]. These findings align with broader epidemiologic observations that females represent a substantial proportion of patients presenting with symptomatic GHOA later in life [28]. A systematic review and meta-analysis evaluating the association between age and sex at onset of GHOA demonstrated that males tend to develop symptomatic GHOA at a younger age compared with females, whereas females more commonly present at an older age with established degenerative disease [28]. Gender-based differences in the underlying diagnoses contributing to GHOA have also been described. Studies have found that males more frequently exhibited secondary osteoarthritis related to prior trauma or instability, whereas females were more commonly diagnosed with primary degenerative GHOA without a clearly identifiable inciting event [28,29,30,31]. These findings are supported by studies evaluating risk factors for degenerative GHOA, which identify male gender, high physical demand, and prior injury as contributors to earlier disease development, while female gender is more frequently associated with age-related degenerative changes [28,30]. Anthropometric and morphologic differences between genders may further contribute to variations in pre-operative disease characteristics. An anthropometric study examining scapular morphology reported gender-based differences in glenoid and scapular dimensions, as well as differences in the prevalence of GHOA between males and females within the studied population [32]. Although primarily descriptive, these findings suggest that structural differences may influence joint loading patterns and degenerative progression.

Collectively, the available literature suggests that males and females with GHOA may differ in age at onset, etiologic pathways, and baseline disease characteristics at presentation; however, these findings remain inconsistent and, at times, conflicting across studies. While some reports indicate that males tend to present earlier and more frequently with secondary or mechanically driven disease and females later with primary degenerative GHOA, other analyses fail to demonstrate clear or reproducible sex-based distinctions.

**Table 2:** Gender differences in GHOA pathology and surgery.

Effect of Gender on Pre-operative characteristics of GHOA	Tran et al., 2022 [27]	-Prevalence similar between sexes (21.4% females vs. 21.0% males). -Females were slightly older on average in older age groups -Prevalence increased with age in both sexes
	Prakash et al., 2024 [28]	-Female sex associated with higher odds of GHOA, though statistical significance was limited. -Age was a stronger predictor than sex.
	Ibounig et al., 2021 [29]	-Advancing age is the major risk factor for GHOA.
	Schoenfeldt et al., 2018 [30]	-Women represented 54% of primary GHOA cases. -Women presented at older age than men. -Distribution of affected arms (dominant/non-dominant/bilateral) similar between sexes.
	Plachel et al., 2023 [31]	-No sex-specific differences in risk factor distribution reported
	Garzón-Alfaro et al., 2024 [32]	-Sex differences not significant in multivariate analysis. -Associated with age and scapular morphology rather than sex.
Effect of Gender on Non-operative management of GHOA	Su et al., 2025 [33]	-Female sex independently associated with failure of nonoperative treatment. -Female patients were more likely to progress to TSA despite conservative management.
Effect of Gender on Outcomes of Shoulder Arthroplasty for GHOA	Mowers et al., 2025 (aTSA) [34]	-Males achieved greater improvements in postoperative ASES and VAS pain scores compared to females. -Females experienced higher rates of postoperative complications and revision surgery
	Stanila et al., 2025 (aTSA) [35]	-Minimal gender-based differences -Males reported less pain at 6 months -Pain, function, range of motion, readmission rates, and revision rates were similar between sexes at final follow-up.
	Okoroha et al., 2019 (aTSA) [36]	-Women began with worse preoperative scores and range of motion. -Post-op improvements did not reach the MCID between genders -Complication profiles differed—Women: higher rates of component loosening and periprosthetic fractures, Men: higher rates of periprosthetic joint infection.
	Stanila et al., 2025 (rTSA) [35]	-Women report higher preoperative pain. -No significant gender-based differences in postoperative pain, function, or range of motion
	Frank et al., 2017 (rTSA) [37]	-Both sexes experienced significant post-op improvement -Female patients achieved lower PROMs and ROM improvement, even after adjusting for age.
	Hochreiter et al., 2023 (rTSA) [38].	-Female sex was identified as a weak independent negative predictor of postoperative objective outcomes -Women demonstrated a significantly higher incidence of intraoperative and postoperative fractures

**Figure 2:**
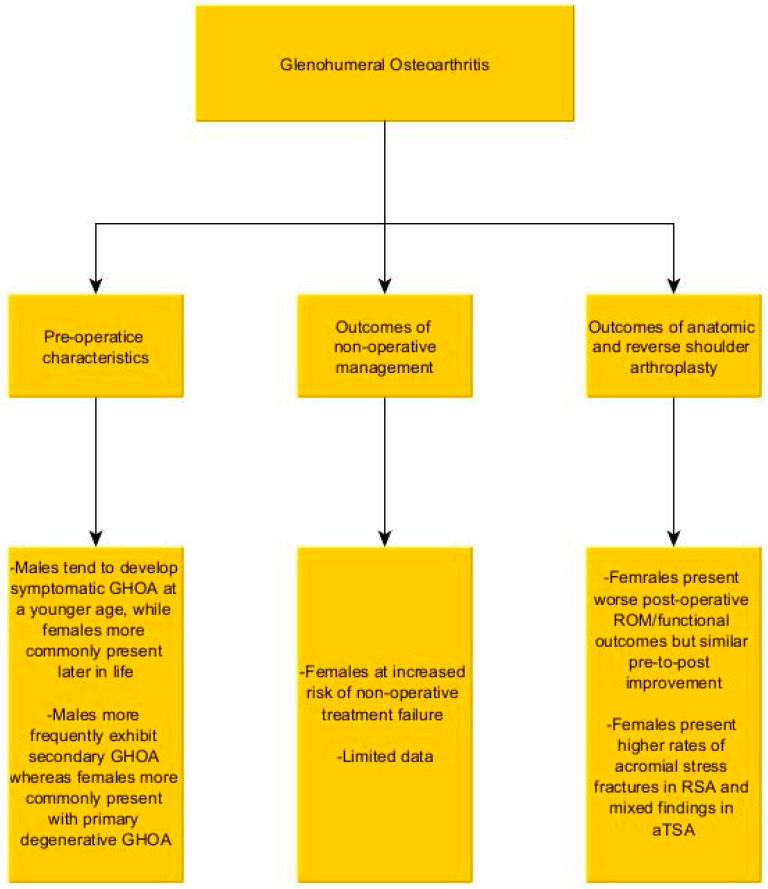
Summary of the literature regarding the impact of gender on glenohumeral arthritis, and its management.

### 4.2. Effect of Gender on Non-Operative Management Outcomes of GHOA (Table 2)

Non-operative management is widely recommended as first-line treatment for patients with GHOA [33,39]. In a prospective cohort study evaluating the effectiveness of non-operative treatment for GHOA, Su et al. demonstrated with a multivariate analysis that female gender was independently associated with failure of non-operative treatment, with females demonstrating a significantly higher hazard of progressing to surgery compared with males [33]. Additional independent predictors of non-operative treatment failure included lack of belief in physical therapy, worsening ASES scores over time, and lower psychological resilience, suggesting that both biological and psychosocial factors influence conservative treatment durability [33]. A contemporary review of non-operative management strategies for GHOA reports that long-term success rates remain limited, particularly in patients with advanced structural disease. However, the review does not report gender-specific differences in response to non-operative management, underscoring the paucity of gender-disaggregated outcome data [39].

Collectively, current evidence suggests that female patients with GHOA may be at increased risk for failure of non-operative management, as demonstrated in prospective cohort data, although the mechanisms underlying this association remain unclear [33]. The limited reporting of gender-stratified outcomes in non-operative studies represents an important knowledge gap.

### 4.3. Effect of Gender on Post-Operative Outcomes of Shoulder Arthroplasty for GHOA (Table 2)

#### 4.3.1. Anatomic Total Shoulder Arthroplasty

Gender-based differences in outcomes following aTSA for GHOA have been increasingly examined [34,35,40]. A systematic review and meta-analysis by Mowers et al. demonstrated that female patients undergoing aTSA experienced significantly worse postoperative functional outcomes, as measured by standardized shoulder outcome scores, compared with male patients [34]. Furthermore, females had higher rates of complications and revision surgery following aTSA, suggesting a clinically meaningful disparity beyond PROMs alone [34]. Studies note that female patients tend to present with worse preoperative functional status, which may partially contribute to observed postoperative differences. Gender-based differences in activities of daily living could also contribute to the different outcome scores observed [37,40]. Of note, baseline physical activity level and functional demand may influence both preoperative presentation and postoperative outcomes following shoulder surgery. Daily activity patterns, occupational workload, and recreational demands can affect baseline strength, pain perception, rehabilitation expectations, and functional recovery. However, physical activity level is inconsistently reported or quantified across studies evaluating gender-based differences in shoulder pathology and surgical outcomes, limiting the ability to draw definitive conclusions regarding its independent effect. As a result, observed differences attributed to gender may in part reflect unmeasured variations in activity level and functional demand rather than gender-related biological factors alone. In contrast, Stanila et al. reported no significant differences between the genders in ROM or pain at final follow-up, with postoperative readmission and revision rates being similar between groups [35]. Similarly, Okoroha et al. found no clinically significant differences in functional scores or ROM, although women demonstrated higher rates of component loosening and periprosthetic fractures, while men experienced more periprosthetic joint infections [36].

Despite these mixed findings, multiple studies consistently note that both male and female patients achieve significant postoperative improvement after aTSA, indicating that the procedure remains effective for GHOA across genders, even if absolute outcomes and complication profiles differ [34,35,36,40].

#### 4.3.2. Reverse Total Shoulder Arthroplasty

RSA is increasingly utilized for GHOA, particularly in older patients or those with glenoid bone loss [38,41,42]. Studies examining RSA outcomes by gender similarly report mixed findings. Stanila et al. found no significant gender-based differences in postoperative pain, function, or ROM following RSA, despite women reporting higher preoperative pain [35]. In contrast, Frank et al. demonstrated that although both genders experienced significant improvement following TSA and RSA, female patients achieved lower final ASES, SST, and VAS scores and showed a smaller magnitude of improvement, even after adjusting for age [37]. Focused analyses of RSA outcomes suggest that the observed differences may be driven by baseline disparities rather than treatment failure. Hochreiter et al. reported that women had worse preoperative and postoperative Constant Scores, but experienced greater relative improvement in pain and internal rotation [38]. Female gender was identified as a negative predictor of postoperative objective outcomes, though differences did not reach the minimal clinically important difference. Importantly, women demonstrated a significantly higher incidence of intraoperative and postoperative fractures, which the authors identified as a major contributor to inferior outcomes [38].

Similar to aTSA, both males and females experience substantial improvements following RSA, with some studies reporting slightly worse patient-reported outcomes among females and others showing no gender differences.

## 5. Shoulder Instability (Figure 3)

### 5.1. Effect of Gender on Pre-Operative Characteristics (Table 3)

Pre-operative characteristics of patients presenting with shoulder instability differ between males and females with respect to epidemiology, mechanism of injury, and baseline functional status. Large cohort analyses demonstrate that males comprise the majority of patients undergoing surgical treatment for shoulder instability, particularly for anterior instability related to traumatic mechanisms and sports participation [42]. Data from the MOON Shoulder Instability cohort showed that females were older at the time of surgical presentation compared with males and were less likely to report a discrete traumatic dislocation event preceding surgery [43]. In the same cohort, females more commonly reported recurrent subluxation episodes or instability symptoms without a clear traumatic onset, whereas males more frequently presented after a single traumatic dislocation. Despite similar distributions of anterior instability diagnoses, females demonstrated a higher prevalence of characteristics consistent with capsular laxity and atraumatic instability patterns [42,43]. Pre-operative PROMs also differed by gender. In the MOON cohort, females reported worse baseline functional scores and greater perceived disability at the time of surgical evaluation compared with males, despite undergoing similar surgical procedures for instability [43]. These findings suggest that symptom burden and functional impairment may be perceived or reported differently between genders prior to operative intervention.

Gender-specific considerations have been further highlighted in studies focusing on female athletes. Reviews of shoulder and elbow pathology in female athletes describe a higher prevalence of atraumatic instability patterns, generalized joint laxity, and neuromuscular control differences in females compared with males, particularly among those participating in overhead or non-collision sports [44,45]. Pathoanatomic evaluation of collegiate female athletes with shoulder instability demonstrated a predominance of soft-tissue pathology, including capsular redundancy and labral injury, with relatively lower rates of significant osseous defects [46]. Hormonal influences have also been proposed as contributing factors to pre-operative instability characteristics in females. A systematic review examining hormonal variation in premenopausal female athletes found associations between estrogen fluctuations and recognized risk factors for shoulder instability, including increased ligamentous laxity and altered neuromuscular control [47]. While causality cannot be established, these findings may help explain the higher prevalence of atraumatic or multidirectional instability patterns observed in female patients.

**Figure 3:**
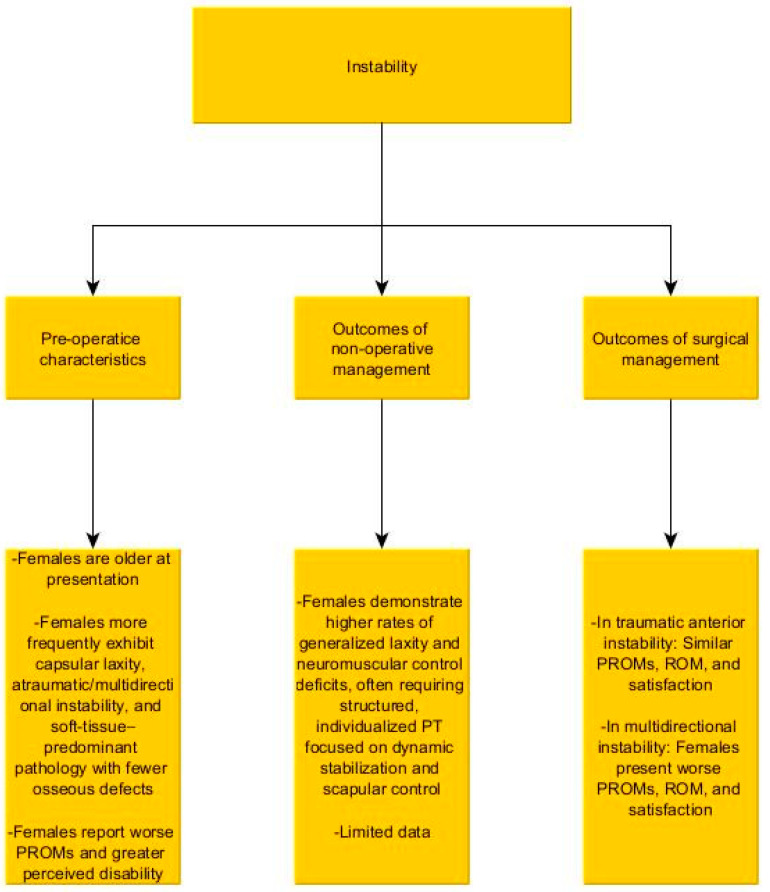
Summary of the literature regarding the impact of gender on shoulder instability, and its management.

**Table 3:** Gender differences in instability pathology and surgery.

Effect of Gender on Pre-operative characteristics of Shoulder Instability	Magnuson et al., 2019 [43]	-Male patients comprised 81.3% of patients undergoing surgery for shoulder instability. -Male patients had a significantly higher rate of traumatic instability -Male patients had higher rates of labral pathology and bone loss. -Female patients had higher rates of capsular laxity. -Females were older at the time of surgical presentation
	Hiemstra & Kirkley et al., 2002 [45]	-Female sex is more commonly associated with non-traumatic instability -Women were less likely than men to undergo surgical stabilization for shoulder instability.
	Patzkowski et al., 2019 [46]	-Majority of instability events in female athletes were traumatic -Predominance of soft-tissue pathology in females -Most female athletes reported multiple instability events
	Wessel et al., 2021 [44]	-Hormonal factors, inflammatory response, and bone mineral density may influence injury patterns in female patients.
	Siddiqui et al., 2025 [47]	-Found associations between estrogen fluctuations and recognized risk factors for shoulder instability
	Wright et al., 2024 [48]	-Identify male sex, younger age, and participation in contact sports as major risk factors for traumatic instability and recurrence.
Effect of Gender on Non-operative Management of Shoulder Instability	Magnuson et al., 2019 [43]	-Male patients comprised 81.3% of patients undergoing surgery for shoulder instability. -Male patients had a significantly higher rate of traumatic instability. -Male patients had higher rates of labral pathology and bone loss. -Female patients had higher rates of capsular laxity. -Females were older at the time of surgical presentation
	Hiemstra & Kirkley et al., 2002 [45]	-Female sex is more commonly associated with non-traumatic instability -Women were less likely than men to undergo surgical stabilization for shoulder instability.
	Patzkowski et al., 2019 [46]	-Majority of instability events in female athletes were traumatic -Predominance of soft-tissue pathology in females -Most female athletes reported multiple instability events
	Wessel et al., 2021 [44]	-Hormonal factors, inflammatory response, and bone mineral density may influence injury patterns in female patients.
	Siddiqui et al., 2025 [47]	-Found associations between estrogen fluctuations and recognized risk factors for shoulder instability
	Wright et al., 2024 [48]	-Identify male sex, younger age, and participation in contact sports as major risk factors for traumatic instability and recurrence.
Effect of Gender on Post-operative outcomes of surgery for Shoulder Instability	Magnuson et al., 2019 [43]	-Female patients had significantly lower preoperative ASES, WOSI, SF-36, and SANE scores.
	Goodrich et al., 2022 [49]	-Male patients had a significantly higher rate of recurrent instability following arthroscopic Bankart repair -No significant sex-based differences in postoperative apprehension.
	Cannizzaro et al., 2020 [50]	-Higher recurrence rates were reported in males (6–37%) compared with females -Sex-specific comparisons of postoperative pain and ROM were inconsistently reported.
	Nguyen et al., 2025 [51]	-No significant sex-based differences in recurrence rates following surgical stabilization. -Comparable postoperative ASES and WOSI scores between sexes.
	Pasqualini et al., 2023 [52]	-Similar recurrence rates and time to recurrence between male and female athletes. -No significant difference in postoperative pain (VAS) between sexes.
	Wessel et al., 2021 [44]	-Surgical management of MDI in females often requires capsular tightening and rotator interval closure. -Female patients with MDI may experience inferior postoperative functional outcomes and higher rates of postoperative instability compared with males.
	Barth et al., 2022 [53]	-Female patients undergoing surgical treatment for MDI are more likely to require rotator interval closure. -Female patients experience higher rates of postoperative subluxation. -Female patients with MDI demonstrate lower postoperative functional outcome scores compared with males.
	Raynor et al., 2016 [54]	-Female patients undergoing arthroscopic pancapsular capsulorrhaphy for MDI demonstrated lower postoperative ASES scores. -Female patients experienced higher rates of postoperative subluxation compared with male patients.

Systematic reviews evaluating risk factors for first-time and recurrent shoulder instability consistently identify male gender, younger age, and participation in contact sports as major risk factors for traumatic instability and recurrence [48]. In contrast, females were more commonly presenting with non-traumatic instability mechanisms and recurrent symptoms related to capsular laxity rather than bony pathology [45,48]. Collectively, these studies demonstrate that although males and females may share similar diagnostic labels, important differences exist in pre-operative presentation, underlying pathoanatomy, and baseline functional status, emphasizing the need for gender-specific assessment and counseling during pre-operative evaluation.

### 5.2. Effect of Gender on Non-Operative Management Outcomes of Instability (Table 3)

Non-operative management is a first-line treatment for select patients with shoulder instability, particularly those with atraumatic or multidirectional patterns. However, gender-specific differences in outcomes following conservative treatment remain poorly defined, as most studies do not report gender-stratified results or include limited female representation [42,48]. Studies indicate that females more frequently present with instability patterns traditionally managed non-operatively, including recurrent subluxations and atraumatic instability, whereas males more commonly present with traumatic dislocations that progress to surgical intervention [42,43,48,55]. As a result, females may be more likely to undergo prolonged or repeated courses of non-operative management prior to surgery [43]. Reviews focusing on female athletes recommend structured physical therapy emphasizing dynamic stabilization, proprioception, and scapular control as the primary non-operative treatment for atraumatic or multidirectional instability [44,45]. These reviews also describe a higher prevalence of generalized joint laxity and neuromuscular control deficits in females, factors that may influence rehabilitation response and necessitate longer or more individualized conservative treatment approaches [44,47]. Furthermore, Cruz-Ferreira et al. demonstrate inferior functional outcomes and higher rates of persistent instability with non-operative management; however, the absence of gender-specific outcome reporting precludes conclusions regarding gender-based differences in response to conservative care [56].

Overall, available evidence suggests that differences in non-operative management outcomes are primarily influenced by variations in instability mechanism, pathoanatomy, and biological factors, rather than gender alone. The lack of gender-disaggregated reporting in non-operative instability studies remains a major limitation. Future prospective investigations should specifically evaluate gender-based differences in rehabilitation response, recurrence rates, and progression to surgical intervention following conservative management of shoulder instability.

### 5.3. Effect of Gender on Post-Operative Outcomes of Instability (Table 3)

Gender-based differences in shoulder instability extend beyond presentation and surgical decision-making and may influence postoperative outcomes. Existing literature evaluating postoperative pain, range of motion, patient satisfaction, and recurrence demonstrates heterogeneity based on instability subtype, surgical procedure, and patient population.

#### 5.3.1. Effect of Gender on Post-Operative Pain Following Surgery for Instability

Data specifically addressing gender-based differences in postoperative pain following surgical stabilization for shoulder instability are limited. In the MOON Shoulder Instability cohort, female patients demonstrated significantly lower baseline PROMs preoperatively, including ASES, WOSI, SF-36, and SANE scores, suggesting greater perceived symptom burden prior to surgery; however, postoperative pain outcomes stratified by gender were not reported [44]. In athletes undergoing arthroscopic Bankart repair, Pasqualini et al. found no significant differences between female and male patients in postoperative pain with both groups demonstrating comparable functional recovery and achievement of clinically meaningful improvements [52]. Similarly, meta-analyses and systematic reviews evaluating anterior shoulder stabilization have not demonstrated consistent gender-based differences in postoperative pain or apprehension, with Goodrich et al. reporting no significant difference in apprehension rates between genders following arthroscopic Bankart repair or open stabilization procedures [49].

In contrast, studies focusing on multidirectional instability suggest that female patients may experience inferior subjective outcomes following surgical intervention. Raynor et al. reported lower postoperative ASES scores and higher rates of postoperative subluxation in female patients following arthroscopic pancapsular capsulorrhaphy, which may indirectly reflect persistent symptoms or discomfort rather than isolated pain measures [54].

#### 5.3.2. Effect of Gender on Post-Operative Range of Motion Following Surgery for Instability

Gender-based differences in postoperative ROM are most prominently discussed in the context of multidirectional instability rather than traumatic anterior instability. Female patients have higher rates of capsular laxity and generalized joint hyperlaxity, which influence both surgical technique and postoperative biomechanics [44,53]. Studies emphasize that surgical stabilization for multidirectional instability in female athletes often necessitates capsular tightening and rotator interval closure, procedures that may result in postoperative reductions in shoulder ROM. In contrast, for unidirectional anterior instability treated with arthroscopic Bankart repair or Latarjet procedures, postoperative ROM outcomes appear comparable between genders [49,51]. Pasqualini et al. similarly reported equivalent functional recovery in male and female athletes when comparing cohorts matched by sport type, suggesting that postoperative ROM outcomes are similar when surgical indications and activity demands are comparable [52].

#### 5.3.3. Effect of Gender on Post-Operative Satisfaction and Recurrence Following Surgery for Instability

Postoperative satisfaction and recurrence rates demonstrate gender-specific patterns that vary by instability type and surgical procedure. For anterior shoulder instability, multiple large-scale studies suggest that recurrence rates following surgical stabilization are similar or lower in female patients compared with male patients. A meta-analysis by Goodrich et al. demonstrated a significantly higher recurrence rate in males following arthroscopic Bankart repair [49]. Cannizzaro et al., similarly, reported higher recurrence ranges in males compared with females across included studies, though heterogeneity limited definitive conclusions [50]. Nguyen et al., conversely, found no significant gender-based differences in recurrence, reoperation, return to sport, or postoperative ASES and WOSI scores across arthroscopic Bankart, open Bankart, and Latarjet procedures [51]. These mixed results emphasize the need for improved gender-disaggregated reporting in future studies. Outcomes following surgery for multidirectional instability appear less favorable in female patients. Female patients undergoing arthroscopic pancapsular capsulorrhaphy were reported to have higher rates of postoperative subluxation and lower functional outcome scores compared with males, despite more frequent use of adjunctive procedures such as rotator interval closure [54]. Additionally, studies evaluating modified Latarjet procedures in female patients have reported lower rates of return to sport compared with published rates in mixed-gender cohorts, suggesting reduced postoperative satisfaction or functional recovery in this population [44].

Overall, existing literature suggests that females undergoing surgical stabilization for anterior shoulder instability may achieve postoperative outcomes comparable to males in terms of satisfaction, functional scores, and recurrence rates; however, these findings are not uniform across studies and should be interpreted with caution. Reported gender-based comparisons vary depending on cohort characteristics, surgical technique, follow-up duration, and outcome definitions. Similarly, while some investigations identify female patients with multidirectional instability as a subgroup with higher rates of postoperative instability and less favorable subjective outcomes, other studies do not consistently reproduce these differences. Consequently, although instability subtype and gender may influence postoperative results, the current body of evidence remains heterogeneous and inconclusive, underscoring the need for more standardized research before definitive counseling or prognostic distinctions can be reliably made.

## 6. Possible Confounding Factors

From a biological perspective, hormonal influences may play a role in musculoskeletal health and pain modulation. Estrogen has been associated with alterations in ligamentous laxity, collagen metabolism, and neuromuscular control, which may partially explain the higher prevalence of atraumatic or multidirectional instability patterns observed in female patients [47]. Additionally, gender-related differences in bone mineral density and muscle mass are well documented, with females generally demonstrating lower baseline muscle strength and higher rates of osteopenia or osteoporosis [25]. These factors may influence preoperative functional status, postoperative rehabilitation capacity, and complication profiles, particularly following shoulder arthroplasty. Differences in shoulder girdle morphology, muscle cross-sectional area, and strength profiles can also affect joint loading, dynamic stability, and functional recovery [57]. Lower baseline strength may disproportionately impact PROMs and satisfaction

Pain perception and reporting also differ between genders and may influence both preoperative assessment and postoperative outcome measures [58]. Females have been shown to report higher pain intensity and greater pain-related disability across multiple musculoskeletal conditions. These differences may reflect variations in nociceptive processing, central sensitization, or psychosocial factors rather than differences in structural pathology alone, potentially contributing to worse baseline scores and lower absolute postoperative outcome measures despite similar relative improvements [58]. Psychosocial and behavioral factors represent additional contributors that are infrequently captured in surgical outcome studies. Differences in activity patterns, occupational demands, expectations of recovery, and psychological resilience may influence symptom reporting, satisfaction, and perceived functional improvement. While some studies suggest that work-related factors and functional demands affect postoperative satisfaction differently between genders, these variables are rarely standardized or adjusted for in outcome analyses.

## 7. Conclusions

The extent and consistency of gender-based differences reported across rotator cuff tears, glenohumeral osteoarthritis, and shoulder instability remain variable within the literature. Although some studies describe differences in clinical presentation and postoperative recovery, these findings are not uniformly observed and are often influenced by heterogeneity in study populations, surgical techniques, outcome measures, and follow-up duration. Reports that females present with greater preoperative disability, lower strength, or higher pain levels are similarly inconsistent, and postoperative recovery patterns appear to vary widely depending on pathology, procedure, and methodological design. Taken together, current data point toward possible gender-related trends rather than definitive differences. These observations emphasize the importance of individualized patient assessment and counseling while highlighting the limitations of the existing evidence base. Future investigations using large, standardized datasets and robust multivariable analyses are needed to clarify whether true sex-based differences exist in presentation, management, and rehabilitation outcomes across shoulder pathologies.

## Data Availability

No new data were created or analyzed in this study.
